# Phenols and GABA_A_ receptors: from structure and molecular mechanisms action to neuropsychiatric sequelae

**DOI:** 10.3389/fphar.2024.1272534

**Published:** 2024-01-18

**Authors:** Sergey A. Menzikov, Danila M. Zaichenko, Aleksey A. Moskovtsev, Sergey G. Morozov, Aslan A. Kubatiev

**Affiliations:** Institute of General Pathology and Pathophysiology, Moscow, Russia

**Keywords:** GABA_A_ receptors, phenols, structures, neuropsychiatric manifestations, molecular mechanisms, ionic plasticity

## Abstract

γ-Aminobutyric acid type A receptors (GABA_A_Rs) are members of the pentameric ligand-gated ion channel (pLGIC) family, which are widespread throughout the invertebrate and vertebrate central nervous system. GABA_A_Rs are engaged in short-term changes of the neuronal concentrations of chloride (Cl^−^) and bicarbonate (HCO_3_^−^) ions by their passive permeability through the ion channel pore. GABA_A_Rs are regulated by various structurally diverse phenolic substances ranging from simple phenols to complex polyphenols. The wide chemical and structural variability of phenols suggest similar and different binding sites on GABA_A_Rs, allowing them to manifest themselves as activators, inhibitors, or allosteric ligands of GABA_A_R function. Interest in phenols is associated with their great potential for GABA_A_R modulation, but also with their subsequent negative or positive role in neurological and psychiatric disorders. This review focuses on the GABAergic deficit hypotheses during neurological and psychiatric disorders induced by various phenols. We summarize the structure–activity relationship of general phenol groups concerning their differential roles in the manifestation of neuropsychiatric symptoms. We describe and analyze the role of GABA_A_R subunits in manifesting various neuropathologies and the molecular mechanisms underlying their modulation by phenols. Finally, we discuss how phenol drugs can modulate GABA_A_R activity via desensitization and resensitization. We also demonstrate a novel pharmacological approach to treat neuropsychiatric disorders via regulation of receptor phosphorylation/dephosphorylation.

## 1 Introduction

Pentameric ligand-gated ion channels (pLGICs), also known as Cys-loop receptors, are the main conduits of chemical neurotransmission in the central nervous system (CNS) (Calimet et al., 2013; Hu et al., 2020). Ion flow via channel gating induces short-term and decremental fluctuations in the transmembrane potential (TMP), allowing dynamic change and control of neuronal excitation and inhibition (E:I) balance in the brain (Gielen and Corringer, 2018). pLGICs have a conserved cylindrical structure in which five subunits are arranged around a central axis (Howard, 2021). Each subunit has a large hydrophilic extracellular domain, which contains a mediator-binding site; four transmembrane domains (TMDs), where TMD2 shapes the ion pore; an intracellular domain; and a short extracellular C-terminal region (Jaiteh et al., 2016; Lara et al., 2020). A dysfunction in pLGICs and subsequent E:I imbalance has been hypothesized to be a key molecular events responsible for neuropsychiatric disorders (Sauguet et al., 2015). Therefore, therapeutic approaches targeting the recovery of the E:I balance are associated with regulating pLGICs.

**FIGURE 1:**
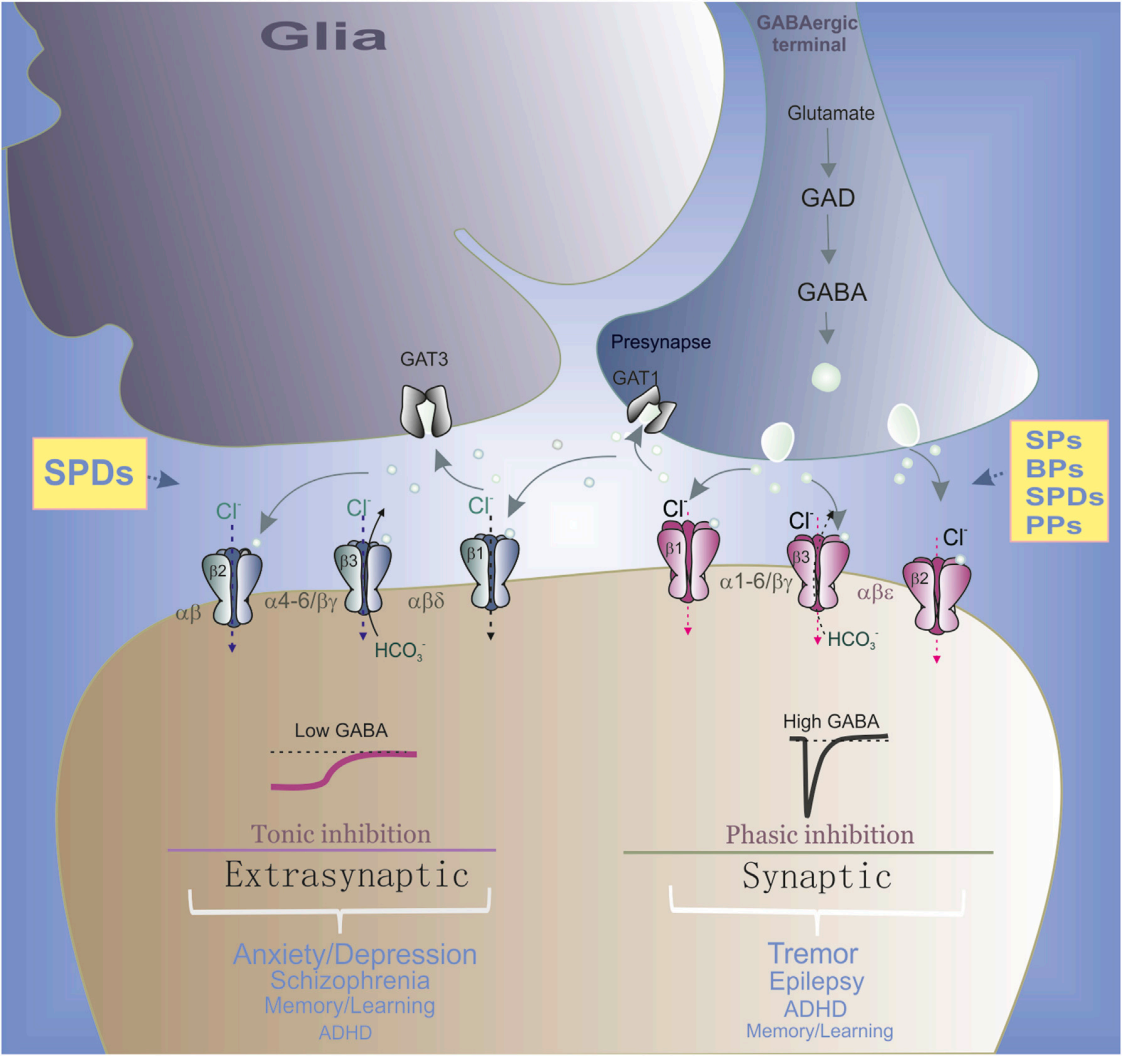
Role of synaptic and extrasynaptic GABA_A_R subtypes in the neurological and psychiatric disorders. In GABAergic terminals, GABA is synthesized from glutamate via glutamic acid decarboxylase (GAD) and then is released from presynaptic neuronal vesicles into the synaptic cleft (circle). GABA reuptake is carried out by GABA transporters (GAT) in both neurons and glia. Specifically, α(1–3)βγ2 subtypes are mainly located in the synapses and mediated the phasic current. Whereas α(4–6)βγ2 or αβε receptor ensembles are partially localized outside the synapse and therefore contributing to both phasic and tonic inhibition. Synaptic GABA_A_R ensembles preferably are involved in the neurological disorders and extrasynaptic subtypes preferably are involved in the neuropsychiatric disorders. All phenols regulate the synaptic GABA_A_R ensembles and SPDs also modulate extrasynaptic subtypes.

As members of the pLGIC family, GABA_A_Rs undergo conformational change upon binding of ligand opening a channel that provides passive permeability for Cl^−^, thereby hyperpolarizing/inhibiting the TMP and reducing neuronal excitability in the adult brain (Ghit et al., 2021). Under certain circumstances (e.g., strong activation), there is also an outflux of HCO_3_^−^ via the receptor channel, resulting in the depolarization/excitation of the TMP (Staley and Proctor, 1999; Kaila et al., 2014). GABA_A_Rs have a pentameric structure formed from 19 identified subunits: α1–6, β1–3, γ1–3, δ, ϵ, π, θ, and ρ1–3 (Zhu et al., 2018). Although the presence of various subunits provides diversity, the most common subunit combinations in the vertebrate brain have tri-heteromeric compositions (2α:2β:1γ), arranged in the following order: γ–β–α–β–α (Sigel and Steinmann, 2012; Borghese et al., 2021). The combination, localization, and functional properties of GABA_A_R subunits are key determining factors in neuronal circuits and the genesis of neurological and psychiatric disorders. Specifically, the α(1–3)βγ2 subtypes are mainly located in the synapses and mediate the phasic current (Figure 1), whereas α(4–6)βγ2 or αβε receptor isoforms are partially localized outside the synapse and therefore contribute to both phasic and tonic inhibition (Feng and Forman, 2018; Sallard et al., 2021). On the contrary, αβ or αβδ GABA_A_R combinations are located extrasynaptically and are involved only in the tonic inhibition of neurons (Sieghart and Savić, 2018). GABA_A_Rs are targets for the action of various drugs, including benzodiazepines, alcohol, barbiturates, neurosteroids, or anesthetics; such pharmacological diversity is determined by the presence of separate binding sites in the structures of the subunits (Sieghart and Sperk, 2002; Olsen and Sieghart, 2009; Sieghart, 2015; Masiulis et al., 2019; Edwards and Preuss, 2022). Many of these drugs act as allosteric modulators of the gating process and stabilize the open state of the receptor pore, potentiating or activating the ionic currents by changing the desensitization processes (with or without orthosteric ligand binding) (Gielen and Corringer, 2018; Kang et al., 2020). Currently, phenols of various origins have attracted special attention because they have great potential for positively modulating GABA_A_Rs and treating diverse diseases, comprising epilepsy, insomnia, anxiety/depression, Parkinson’s disorder, autism, or schizophrenia (Yamaura et al., 2016; Hu et al., 2020). Although analyses of the structure and function of GABA_A_Rs (Phulera et al., 2018; Zhu et al., 2018) and their binding with various drugs have expanded our understanding of their pharmacological profiles (Sieghart, 2015; Puthenkalam et al., 2016; Engin et al., 2018; Liu et al., 2018; Masiulis et al., 2019), the molecular events via which phenols interact with specific sites on GABA_A_Rs remain poorly understood, although models are beginning to emerge.

**FIGURE 2:**
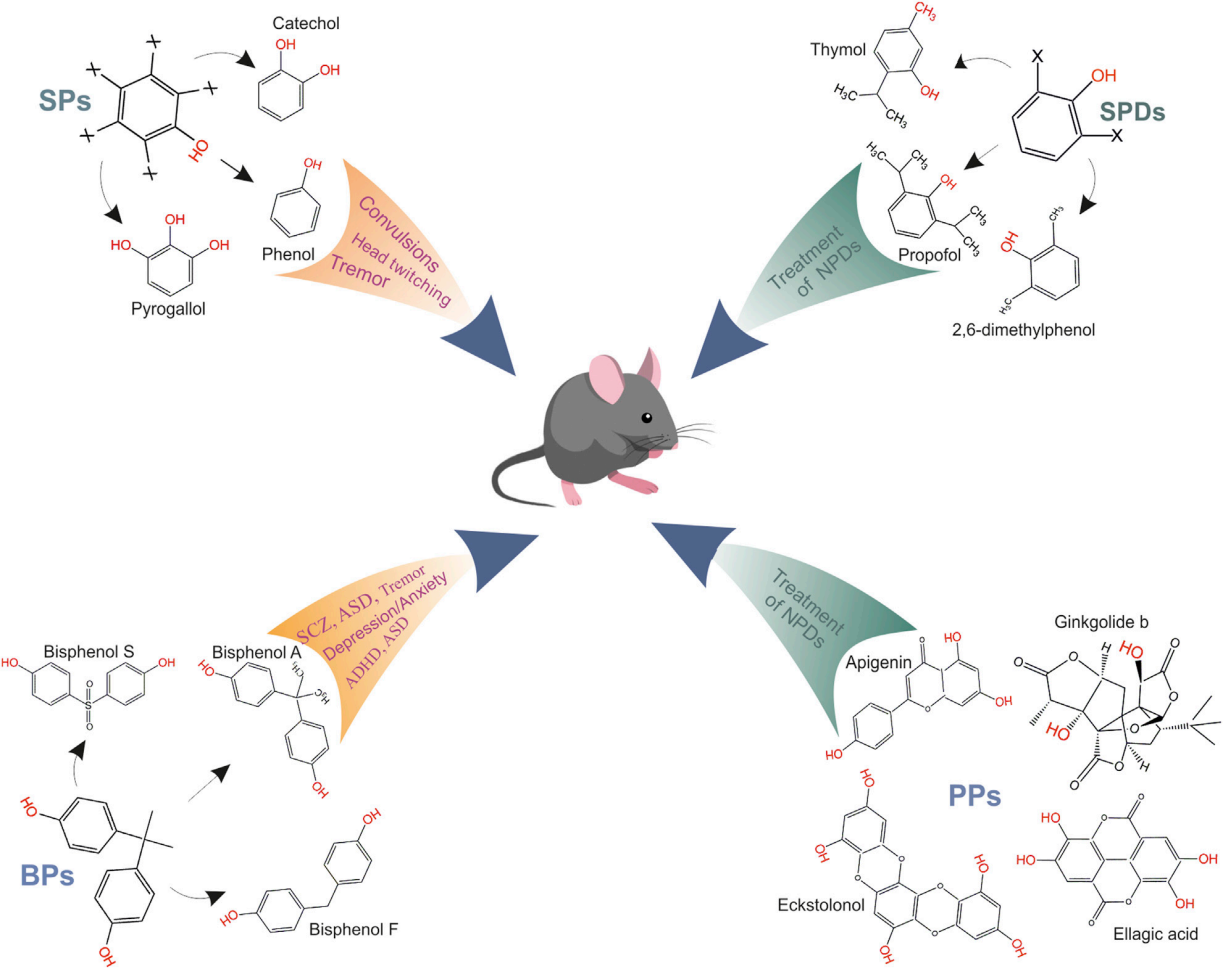
Role of various phenol groups in appearance of neuropsychiatric disorders. SPs mediate head twitching, tremor and epileptic seizures (sceleton structure of SPs, where x is possible presence of a hydroxyl group and representatives of group are phenol, catechol or pyrogallol); BPs mediate the tremor, anxiety/depression, ADHD, schizophrenia and memory/learning, ASD (sceleton structure of BPs and representatives of group are bisphenol A, bisphenol S or bisphenol F); SPDs are general anesthetics and use for treatment of neuropsychiatric disorders (sceleton structure of SPDs, where x is possible presence of a substituting group and representatives of group are propofol, thymol or 2,6-dimethylphenol); PPs have neuroprotective effects and were used for treatment of neuropsychiatric disorders, and representatives of group are apigenin, ginkgolide b, eckstolonol, ellagic acid.

Phenols display a great variety of structures, ranging from simple phenols (SPs) and their derivatives (SPDs) to bisphenols (BPs) and complex natural polyphenols (PPs) (Figure 2). Natural phenolic compounds’ features and roles in GABA_A_R regulation have been extensively discussed in recent reviews (Cichon et al., 2020; Rahman et al., 2021; Ríos et al., 2022). However, the chemical and structural variability of phenols suggests the presence of various binding sites on GABA_A_Rs, allowing them to be activators, blockers, or allosteric ligands of GABA_A_R activity (Al Mamari, 2021). The presence of several opposing characteristics determines interest in SPDs and PPs as independent modulators of GABA_A_Rs function: their potential pharmacological use as anesthetic, anxiolytic, anticonvulsant, sedative, or muscle relaxant drugs (Sauguet et al., 2015) and their ability to exert a neuropathological effect upon accumulation at high concentrations, prompting the search for the optimal phenol structure with the least negative effect (Mohammadi et al., 2001). Moreover, the interest in natural phenols is associated with the need to find new active chemicals different from other pharmacological drugs, avoiding several undesirable effects, such as tolerance, abstinence, dependence, and memory disorders. The ability of SPs and BPs to cause neuropsychiatric disorders such as head twitching/tremors and seizures (Figure 2) (Spencer et al., 2007; Menzikov and Morozov, 2019), attention-deficit/hyperactivity disorder (ADHD), depression, anxiety, and schizophrenia (Bowman et al., 2015; Perera et al., 2016) require a clarification of the molecular mechanisms underlying the appearance of these disorders (Detyniecki, 2022). Literature evidence supports the potential action of various phenols as positive modulators of GABA_A_Rs, which favors their use in developing new therapeutic drugs (Lasoń and Leśkiewicz, 2013; Cordeiro et al., 2022). However, additional investigation is needed to determine the minimal structural complexity needed for phenolic compounds to activate GABA_A_Rs.

This review focuses on the GABA_A_ergic deficit hypotheses during neuropsychiatric disorders induced by various phenols and the molecular mechanisms underlying their interaction with GABA_A_Rs. We analyzed the structures of various phenols and how they induce different neuropsychiatric manifestations, from negative (by SPs or BPs) to positive effects in the CNS (by SPDs and PPs). We also highlighted and discussed the role of phenols in the modulation of GABA_A_Rs by analyzing their extra- and intracellular binding sites. Next, we analyzed the role of GABA_A_R subunits in phenol-induced neuropsychiatric disorders and the molecular mechanisms underlying their modulatory effects. Finally, based on current literature, we discussed how phenols can regulate GABA_A_R activity via processes of the desensitization and resensitization. Overall, this review demonstrated novel pharmacological approaches when using phenolic substances to treat diseases via regulation of the receptor phosphorylation/dephosphorylation.

## 2 Phenols and neuropsychiatric disorders

**FIGURE 3:**
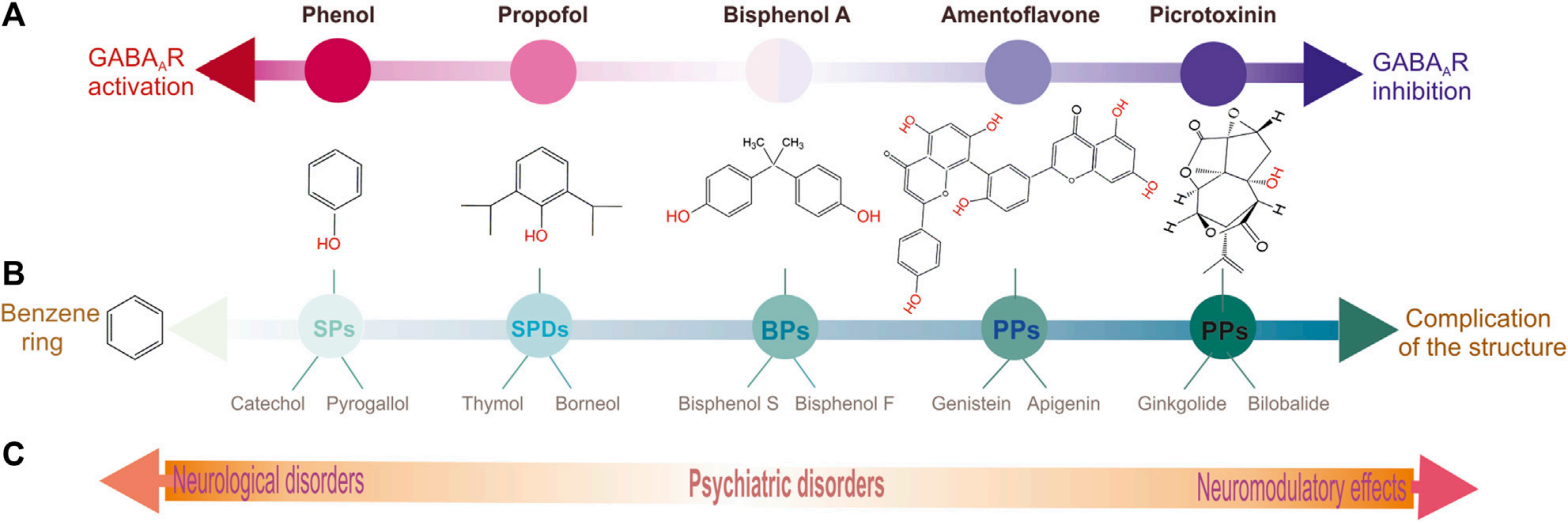
Schematic representation distributing of various phenol groups by their biological and structural peculiarities. **(A)** Chemicals distribution in according to their type of action on GABA_A_Rs: left is activation, right is inhibition and in the middle is intermediate state. **(B)** Chemicals distribution in according to complexity of their structure. **(C)** Chemicals distribution in according to their the role in the neuropsychiatric sequelae.

Phenols are a class of aromatic chemicals characterized by a hydroxyl (-OH) group attached directly to a carbon atom of a benzene ring (Al Mamari, 2021). Phenols are among the largest and most widely distributed group of aromatic hydrocarbons. Phenols can be divided into subgroups according to their structural and biological characteristics since they can produce both beneficial and harmful effects on living systems depending on the phenols’ structural features and doses (Figure 3). Indeed, despite the wide use of phenol compounds in various industries, their biological significance is usually considered in the context of their effects on the environment and on human health (Li, 2017; Tufarelli et al., 2017). Human and animal health, especially, is adversely impacted through lifetime exposures to environmental stressors, such as chemicals present in the water, air, and food. Crucial sources of environmental pollution are phenolic compounds (Vermerris and Nicholson, 2008). Moreover, many studies have demonstrated that phenols cross the blood-brain barrier (BBB) and can exert positive or negative health effects on the brain (Pletz et al., 2016; Velásquez-Jiménez et al., 2021). For example, when phenols of various origins enter the bodies of animals and humans, these compounds can have undesirable and beneficial neuropsychiatric consequences (Tufarelli et al., 2017). Although, based on their neurotoxicological features, phenols are essentially distinct from each other, there are not only visible differences but also similarities in their influence on the CNS (Giri et al., 2016; Ismail and Mahboub, 2016).

### 2.1 Simple phenols

Simple phenols (SPs) are molecules with one or several -OH groups attached to a benzene ring, which is their basic skeleton (Figure 2). The simplest representative compound of the SP class is phenol (C_6_H_6_O), also known as benzenol or carbolic acid, which contains a single hydroxyl group. Within the SP group, catechol, resorcinol, pyrogallol, and phloroglucinol contain either two or three hydroxyl groups (Figure 3). SPs can either be natural or artificial compounds; phenol is found in coat tar, while pyrogallol, resorcinol, catechol, and phloroglucinol are found in plants. SPs are ubiquitous chemicals used in manufacturing fragrances, pharmaceutical drugs, and flavor enhancers and are essential for producing polycarbonates, epoxies, bakelite, nylon, detergents, herbicides, and fur dyes. However, the main use of phenol, which accounts for two-thirds of its production, involves its conversion to precursors that are used in plastics. Environmental exposure to phenols occurs in various ways via industrial processes, such as during pyrolysis and in chemical effluents. Many SPs are corrosive to the eyes, skin, and respiratory tract, and long-term and/or repeated exposure to these substances may harm the liver and kidneys. SPs may also cause harmful effects on the CNS and heart, resulting in arrhythmia, seizures, and coma. Phenol-induced neurological disorders include consecutive manifestations of several neurological symptoms, such as head twitching, tremors, epileptic seizures, coma, and even death (Hodson, 1985; Rice et al., 1997; Todorović, 2003; Giri et al., 2016; Menzikov and Morozov, 2019).

*Tremors* are neurodegenerative movement disorders (Gironell, 2014). Tremor-related pathologies are classified according to their characteristics, and their clinical manifestations include motor symptoms (including bradykinesia, rigidity, postural instability, and/or resting tremors) and/or non-motor symptoms (Błaszczyk, 2016). Phenol-induced effects manifest primarily as head twitching and muscular tremors followed by the spreading of such manifestations to other body parts (Spencer et al., 2007). The magnitude of head twitching/tremors depends on the compound dose, and the maximum effect occurs 5–10 min after exposure (Menzikov and Morozov, 2019; Itoh, 1995; Windus-Podehl et al., 1983).

*Epilepsy* comprises a group of neurological disorders with neurobiological, cognitive, and psychological symptoms characterized by repetitive epileptic seizures with a range of etiologies and comorbidities (Hirose, 2014; Kaila et al., 2014; Fei et al., 2020). Epilepsy is mostly considered an impairment of the excitation: inhibition (E:I) balance due to alterations in synaptic neurotransmission. Phenol-induced tremors are accompanied by severe seizures which are dependent on the phenol dose (Windus-Podehl et al., 1983; Itoh, 1995). GABA_A_ergic ligands (phenobarbital or picrotoxin) have been shown to restore phenol-mediated behavioral changes (Menzikov and Morozov, 2019).

### 2.2 Simple phenol derivatives

Simple phenol derivatives (SPDs) are simple phenols on which one or more hydroxyl groups are substituted with methyl, amino, or halogen groups as shown in Figure 2) (Al Mamari, 2021). Propofol and thymol, which are general anesthetics, are well-known as representative SPDs. Propofol induces and maintains general anesthesia and sedation in human adults and is administered via injection into a vein. It takes approximately 2 min to reach its maximum effect and typically lasts 5–10 min. This drug may cause a decrease in the processes associated with consciousness and memory and can also induce euphoria, hallucinations, and disinhibition. Propofol is also used to treat epileptic disorders if other drugs are ineffective (Meyer et al., 2006).

The use of SPDs is limited because of the severe neuroexcitatory symptoms that occur at high concentrations of these compounds that accumulate in the brain. Propofol-induced convulsions are related to the suppression of inhibitory circuits in the CNS (Islander and Vinge, 2000; Pantelakis et al., 2021). SPDs can cause excitatory phenomena, such as epileptiform movement or seizures (Moore et al., 2021). Moreover, among the various SPDs studied, only OH-substituted naphthalenes were active in inducing myoclonic convulsions in rodents, indicating that the phenolic hydroxyl group is an essential requirement for inducing these neurological manifestations (Haeseler and Leuwer, 2002).

### 2.3 Bisphenols

Bisphenols (BPs) are a large group of compounds that contain two phenolic rings linked by a bridge that is formed by carbon and other chemical structures (Figure 2). BP compounds are derivatives of bisphenols for which the capital letter after that acronym is used to express the reactant atom/component within its chemical structure. For example, the A in bisphenol A (BPA) stands for acetone, the S in bisphenol S (BPS) denotes sulfur trioxide, and the F in bisphenol F (BPF) stands for formaldehyde. Other letters representing other compounds are also used as shown in Figure 3 (Qiu et al., 2019). BPs are widely produced synthetic chemicals and are typically used to manufacture polycarbonate plastics, including dental composites, bottles, and sealants (Yoo et al., 2020). BPs are also used to add strength and resilience to materials and are found in many products, such as toys, water supply pipes, and medical tubing (Brown, 2009; Li et al., 2023). BPs are ubiquitous within the environment and have become a health threat due to their neurotoxicological effects on animals and humans. Many studies on the biological and neurotoxicological actions of BPs on the CNS have focused on their estrogenic action as an endocrine disrupter (Choi et al., 2007; Braun et al., 2009; Marques et al., 2021; Rebolledo-Solleiro et al., 2021).

BPA is a contaminant of utmost concern because it is an endocrine disruptor that may also affect neurodevelopment in children (Brown, 2009). The primary route for introducing BPA in humans is via the consumption of food products that have been in contact with materials containing this chemical. BPA is a major component of epoxy and polycarbonate resins, which are widely used as ingredients in protective coatings on food containers and as adhesives used in packaging products. Low doses of BPA induce cancer, an increase in body weight, adverse effects on the male reproductive tract, and long-term changes in brain structure and function. Neurocognitive effects, such as hyperactivity, aggression, and impaired learning have also been demonstrated in animals treated with BPA. Although other BP compounds have been used to replace BPA, evidence that such replacement compounds show similar toxicity to BPA has been reported (Naderi et al., 2022). However, a comprehensive assessment of these replacement compounds still needs to be done.

Invertebrate and vertebrate animal models have shown that exposure to BPA can adversely affect multiple aspects of neuronal development, including neural stem cell proliferation, differentiation, and synaptic plasticity. Investigations on animal models have revealed that BPA treatment considerably concurrently affects behavioral endophenotypes, including changes in locomotor activity, induction of anxiety-like behavior, and learning/memory deficits (Stump et al., 2010; Bowman et al., 2015; Mornagui et al., 2019; Welch and Mulligan, 2022).

*Tremor −* Some reports demonstrate that BPA exposure may affect the development of Parkinson’s disorder (Landolfi et al., 2017; Li et al., 2023). Administration of BPs led to a worsening of tremors in mice via NRF2/HO-1 signaling (D’Amico et al., 2022). BPA (≤1 mM) was found to induce oxidative stress and impair mitochondrial and cellular metabolic activity in the heads of flies (*Drosophila melanogaster*), causing the development of a Parkinson ’s-like disease (Musachio et al., 2020). In addition, high concentrations of BPA produced changes in the expression of tremor marker proteins in the zebrafish brain (Olsvik et al., 2019).

*ADHD* is a childhood neuropsychiatric disorder characterized by behavioral deviations, including hyperactivity, attention deficit, and impulsivity (Kwon et al., 2017). However, despite the availability of knowledge regarding the diverse mechanisms of ADHD, its pathogenesis is not fully understood, so correct early diagnosis and provision of accurate clinical management are challenging (Kessi et al., 2022). Recent papers have speculated a strong correlation between BPA treatment and behavioral/cognitive dysfunction in children with ADHD (Tewar et al., 2016; Rochester et al., 2018; Yoo et al., 2020; Subramani et al., 2023). Life-course analysis revealed that a diet containing BPA led to an increase in energy expenditure in mice and was associated with hyperactive and lean phenotypes (Anderson et al., 2013). In another study, BPA administration (0.01–1 μM) led to larval hyperactivity or learning deficits in adult zebrafish (Saili et al., 2012). Interestingly, exposure to β-estradiol (0.1 μM) also led to larval hyperactivity. In a recent paper, it was demonstrated that treating *D. melanogaster* embryos with BPA (1 mM) induced pathological developmental changes in behavior, such as incompatibility in social interactions and hyperactivity, which manifest as an enhancement of locomotion in open field tests and aggression episodes (Musachio et al., 2021).

*Anxiety* is characterized by worry, apprehension, and specific somatic, neurocognitive, and behavioral manifestations (Hu et al., 2023). *Depression* includes emotional symptoms, such as persistent anhedonia, sadness, reduced interest of the person in their environment, and psychomotor changes (Xing et al., 2012). Several studies have suggested that gestational exposure to BPs may lead to neurobehavioral problems during childhood and impaired socio-cognitive development and associated mechanisms. In a study by Perera et al. (2016), BPA administration was coupled with symptoms of anxiety and depression more frequently in boys than in girls aged 10–12 years. Many studies have also shown that long-term application of BPA and its phenolic analogs induces various sex-specific anxiety and depression symptoms in rodents (Xu et al., 2015; Xu et al., 2012; Hong et al., 2013; Kumar and Thakur, 2017; Wiersielis et al., 2020). Specifically, mice exposed to low doses of BPA in their drinking water during the pre- and post-natal periods showed anxiety-related behaviors (Sasaki et al., 2023). In another study, BPA administration elevated anxiety behaviors in F0 rats (Fan et al., 2018). Paternal exposure of F1 rats also led to an increase in anxiety behaviors in F1 females and aggravated depressive behaviors in both sexes. A recent study revealed that administration with low concentrations of BPA and BPS in early life caused an elevation in anxiety-like behavior in zebrafish (Naderi et al., 2022). In contrast, exposure to higher concentrations of BPA resulted in social deficits and impaired object recognition memory. Additionally, co-exposure to an aromatase inhibitor antagonized the BPA- and BPS-induced effects on anxiety levels and social behaviors in zebrafish larvae. Co-application with an estrogen receptor antagonist restored normal recognition memory. The authors speculated that BPA and BPS affect social and cognitive functions via different mechanisms.

*Schizophrenia* is a disorder with an unknown etiopathogenesis. It manifests as disturbed behavior characterized by delusions, hallucinations, abnormal mental functions, disorganized thinking, and impaired cognitive function. Although several hypotheses have been proposed, the etiology of schizophrenia remains unclear. Accumulating data demonstrate that native endocrine disrupters are associated with this disease (Bowman et al., 2015). Moreover, the pathology of schizophrenia has been associated with impairments in mitochondrial function, energy expenditure, and oxidative stress (Chiapponi et al., 2016).

*Memory* and *learning* refer to how an individual acquires, encodes, stores, and retrieves information (Rebolledo-Solleiro et al., 2021). The hippocampus is one of the main structures involved in various cognitive functions, including memory and learning. Several studies have reported that BPA could influence learning and memory, which sometimes appear to be sex-dependent. For example, male rats that received high concentrations of BPA during adolescence showed deficient spatial memory and anxiety in adulthood (Bowman et al., 2015; Wu et al., 2020; Hu et al., 2023). In contrast, other studies report that rats exposed to BPA (25 μg/kg/day) had altered spatial learning as determined using a Morris water maze; however, this observation was only noted in females (Hass et al., 2016). However, some authors have not found significant differences in spatial learning between male and female deer mice (Jašarević et al., 2013). It was also reported that mice showed decreased alternation behavior in a Y-maze after treatment with BPA at doses of 100 and 500 mg/kg/day (Rebolledo-Solleiro et al., 2021). These results indicated working memory impairment. BPA-treated mice also showed a decrease in novel object recognition as indicated by a reduction in central locomotion and frequency in the central zone (Tian et al., 2010). Mice of both sexes that received BPA (0.5–5,000 μg/kg/day) presented increased anxiety, impaired spatial memory, and reduced dendritic spine density in the CA1 region of the hippocampus and medial prefrontal cortex (Xingyi et al., 2013; Zhang et al., 2019; Zhang et al., 2020). Moreover, low-dose maternal BPA application caused significant impairment in the learning/memory capabilities of F1 male mice but not in the F2 generation. Wang et al. reported adverse effects of BPA on the CNS, especially learning and memory (Wang et al., 2014). BPA has been shown to cause adverse effects on the synaptic structure of pregnant Sprague-Dawley rats, resulting in a widened synaptic cleft, thinned postsynaptic density, and unclear synaptic surface. In addition, environmental exposure to BPA may impair childhood behavior and learning development. For example, urinary BPA levels are negatively coupled to the learning quotient based on the Learning Disability Evaluation Scale (LDES) as described in several studies (Hong et al., 2013).

*Autism* is characterized by several behavioral features, including social deficits, impaired communication, and repetitive behaviors with sex-specific manifestations (Stein et al., 2015). Pre-natal exposure to high BPA concentrations is thought to elevate the risk of developing autism. Recent studies have identified candidate autism-related genes responsible for the sex-specific prenatal action of BPA (Thongkorn et al., 2023). Molecular docking analysis of BPA and autism-related transcription factors revealed targets for BPs (Kanlayaprasit et al., 2021). In addition, it was found that prenatal BPA administration caused an elevation in neurite length, the number of primary neurites, and the number of neurite branches but diminished the size of the hippocampal cell body in both sexes. Several epidemiological studies demonstrated the association between prenatal BPA application and autism (Kanlayaprasit et al., 2021); however, other studies did not find any association between prenatal BPA application and neuropathology (Hansen et al., 2021).

### 2.4 Polyphenols

Polyphenols (PPs) are a class of compounds with a polyphenol structure and one or more -OH groups attached directly to several benzene rings (Figure 2). Natural phenols include two types of compounds: 1) flavonoids and 2) non-flavonoids (Balland et al., 2022; Ríos et al., 2022). Flavonoids consist of flavones, flavonols, flavonones, isoflavones, and anthocyanins, which differ via their hydrogenation status and the identity of their heterocyclic substitutions (Figure 3). Other PPs include phenolic acids, terpenes (mono-, di-, tri-, and sesquiterpenoids), stilbenes, phlorotannins, and lignans.

Natural polyphenols are secondary metabolites ubiquitous throughout the plant kingdom. PPs are formed in variable amounts depending on the taxonomic group (family or species) and plant parts, such as roots, stems, flowers, and fruits. In plants, these chemicals play a key role in many vital physiological processes, such as lignification, pigmentation of flowers and fruits, and pollination, and also act as growth factors. They also govern plant responses to environmental stress, such as protection from excess ultraviolet radiation, nutrient deficiency, and drought stress, and act as chemical defenses against herbivores, insects, and microbial pathogens. Natural phenolics are important constituents of many edible and medicinal plants and are widely used in the food industry as flavorings, antioxidants, and antibacterial agents. Indeed, PPs have attracted significant attention due to their wide presence in our daily diet and, more importantly, their antioxidant properties (Rahman et al., 2021; Karioti et al., 2016). Although the relationships between food phenolics and health are not yet fully understood, human epidemiological studies demonstrate that consuming food rich in polyphenols might be beneficial, especially for age-related disorders, such as cardiovascular and neurodegenerative diseases. While various studies have investigated extracts and essential oils as potential sources of active agents in animals and humans, other authors studied their possible pharmacological potential *in silico*. For example, flavonoids have antimicrobial, hepatoprotective, cardioprotective, anti-inflammatory, neuroprotective, antiviral, and anticancer properties (Rahman et al., 2021). Of all tested compounds, phenolics were of greatest interest, especially flavonoids and certain tannins. At present, convincing evidence that the mechanisms by which flavonoids exert their pharmacological effects are not simply due to their redox properties but their capability to bind directly to target proteins or peptides that regulate different cellular functions exists (Karioti et al., 2016).

Natural phenols can be beneficial to health because they cause a reduction in the risk of developing neurological and psychiatric diseases (Vermerris and Nicholson, 2008; Szwajgier et al., 2017). Many authors have reported the neuroprotective role of various phenolic acids in controlling epilepsy and ameliorating anxiety and depression, imbalance after traumatic brain injury, hyperinsulinemia-induced memory impairment, and Parkinson’s disorder (Szwajgier et al., 2017; Fasipe et al., 2020; Cordeiro et al., 2022; De Vries et al., 2022; Dong and Huang, 2022). Ellagic acid has antioxidant, anti-inflammatory, and neuroprotective properties (Ríos et al., 2022). Several recent reviews have discussed the antidepressant effects of compounds, such as amentoflavone, apigenin, chlorogenic acid, curcumin, and ferulic acid (Pathak et al., 2013; Wang et al., 2022; Winiarska-Mieczan et al., 2023). In another study, *Nigella sativa* oil produced a positive motor coordinative effect on phenol-mediated essential tremors in a mouse model (Folarin et al., 2020). Recent studies have also suggested using plant-derived polyphenolic compounds for antiepileptic treatment. In particular, extracts from *Urtica dioica* Linn. root or *Lactuca serriola* (Asteraceae) can cause antiepileptic effects in pentylenetetrazole-induced seizure models (Loshali et al., 2021). Among flavonoids, chrysin, resveratrol, baicalein, quercetin, and rutin produced significant anti-seizure activity or mild sedative and anxiolytic effects (Ullah et al., 2022; Karioti et al., 2016). Thus, flavonoids can prevent neuronal excitability, suggesting their potential use as an adjunctive therapy for treating epilepsy. However, although natural phenols possess diverse biological properties, the molecular mechanisms underlying their effects on the CNS remain unknown.

## 3 Phenols and GABA_A_Rs

Although the neurotoxic actions of aromatic hydrocarbons on animals and humans manifest themselves at the organismic, cellular, and molecular levels, they primarily affect the CNS. Therefore, understanding where and how phenol and phenol derivatives interact with subcellular brain structures remains relevant at the present time. Moreover, substances with a wide range of action on the organism can have additive and/or neutralizing influences on each other (Hellström et al., 2016). The research concerning the brain structures that are specific and highly sensitive to a wide row of phenolic compounds is important for obtaining a better understanding of their neuropsychiatric mechanisms action. Many studies have confirmed that their neurotoxicity effects are associated with modulating pentameric ligand-gated ion channels (pLGIC) activity. Specifically, data demonstrate that phenolic compounds selectively interact with GABA_A_Rs and prove to be modulators by inducing negative or positive effects on the CNS (Figure 3).

### 3.1 Simple phenols

**FIGURE 4:**
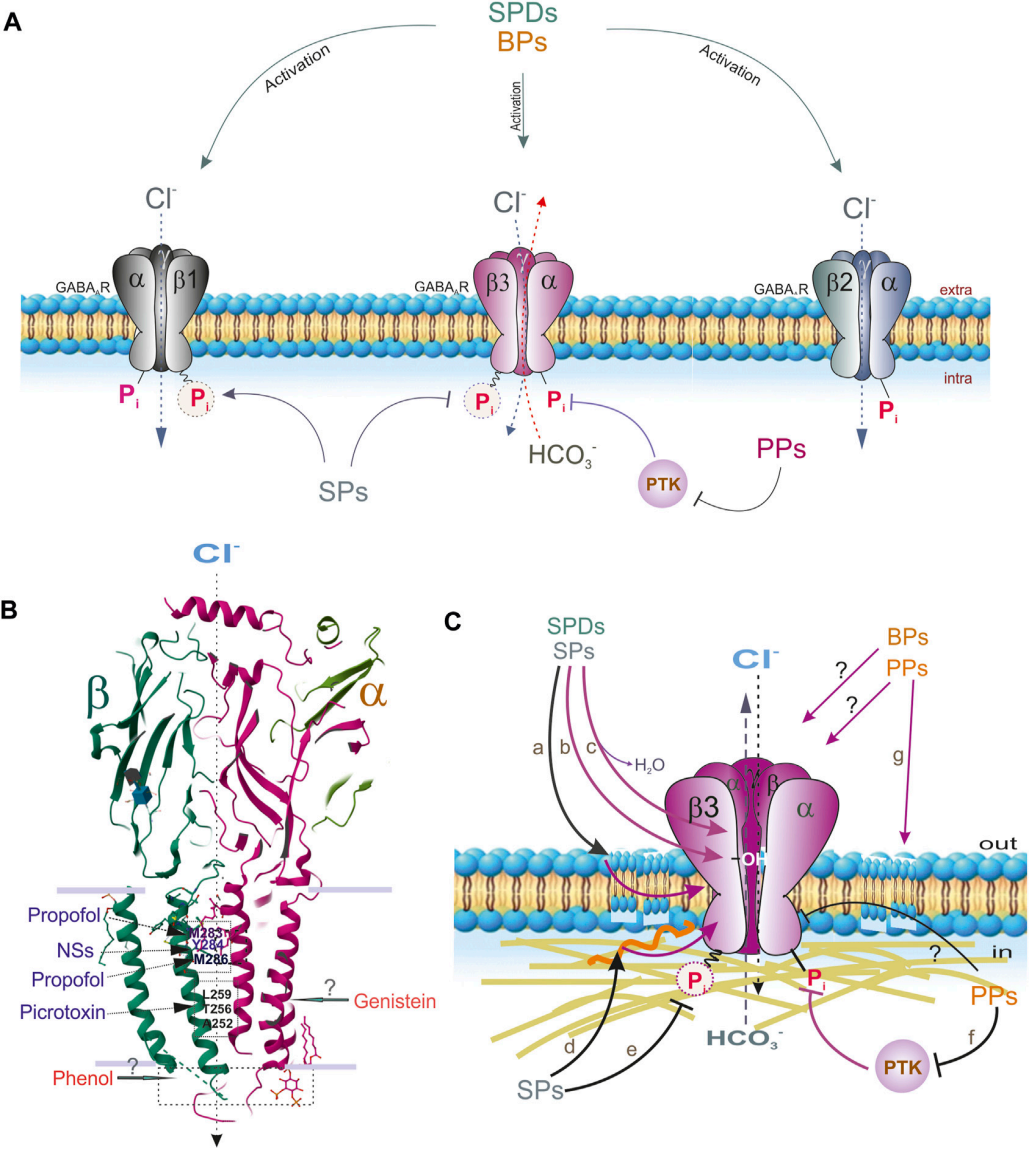
Extra- and intracellular effects of different phenols on the GABA_A_R subtypes containing β1, β2 or β3 subunits. **(A)** SPDs interact with all receptor subtypes. SPs interact with β3 and β1-containing GABA_A_R subtypes via binding with ATP-hydrolyzing site. PPs directly inhibit GABA_A_R function or via PTK way. **(B)** Sites binding of propofol, picrotoxin or NSs with GABA_A_R β and α subunit (Protein Data Bank, 4COF). Sites binding of phenol or genistein are not known. **(C)** The suspected molecular mechanisms interactions of SPs or SPDs on the GABA_A_Rs: a) and g) via membrane lipids modification; b) via H-bond formation with OH-groups; c) via displacing of water; d) via cytoskeleton disturbance; e) via changes of the formation of high-energetic acyl-phosphate bond; f) via the processes of phosphorylation by PTKs (or other protein kinases) or direct inhibition of the receptor function.

Early studies demonstrated heterogeneous data regarding the mechanisms underlying phenol-induced NDs. For example, the effects of catechols on neuromodulator uptake in rat brain slices showed that low catechol concentrations (10 μM) had little effect on the release of acetylcholine, noradrenaline, or GABA but produced an increase in aspartate release (Minchin and Pearson, 1981). In contrast, a high catechol concentration (100 μM) led to an inhibition of acetylcholine and GABA release. Ducis et al. have shown that phenol affects the peripheral benzodiazepine receptors on astrocytes (Ducis et al., 1990). However, it is likely that the effects were directed at a translocator protein (TSPO), also known as a peripheral benzodiazepine receptor, which is a transmembrane protein located on the outer mitochondria membrane in the glial cells of the brain (Lee et al., 2020). Several authors have shown that 10 mM phenol activates oocyte-expressed GABA_A_R-mediated Cl^−^ currents by displacing water as shown in Figure 4A (Brosnan and Pham, 2018). Both *in vitro* and *in vivo* studies recently demonstrated that phenol has a biphasic action on the Cl^−^-ATPase activity of β3-containing GABA_A_Rs isolated from rat and fish brains with receptor activation at low concentrations and inhibition at high concentrations (Menzikov, 2018; Menzikov and Morozov, 2019). Moreover, at low doses (<100 μM), phenol was found to inhibit Cl^−^/HCO_3_^−^ ATPase function and activate Cl^−^ transport via GABA_A_Rs that had been purified from the rat brain (Figure 3).

### 3.2 Simple phenol derivatives

Primary alkylphenol-injectable anesthetics are potent drugs with a high affinity to recombinant GABA_A_Rs; at high doses, these anesthetics may also directly potentiate receptors as shown in Figure 4A (Weir et al., 2017; Borghese et al., 2021). General anesthetics can induce different changes in the GABA_A_R αβγ and αβδ complexes in either their activation or desensitization states (Feng and Forman, 2018). Structure–activity studies using recombinant α1β2γ2 GABA_A_Rs addressing the direct agonist effects of SPDs revealed that only chemicals with both unsubstituted phenolic hydroxyls attached directly to the benzene ring or a methyl or isopropyl group inserted in the ortho position relative to the -OH group induced inward Cl^−^ currents (Mohammadi et al., 2001). In addition, thymol (one isopropyl group at the ortho-position) was slightly more potent than 2,6-dimethylphenol; however, propofol possesses the highest potency for GABA_A_Rs.

The propofol binding sites on the GABA_A_R have been identified via site-directed mutagenesis (Shin et al., 2018), substituted cysteine modification protection (Forman and Miller, 2016), or photo-affinity labeling (Olsen and Li, 2011; Jayakar et al., 2014). Specifically, results of modeling, photolabeling, and/or functional studies demonstrated two propofol binding domains on GABA_A_Rs (Krasowski et al., 1998; Krasowski et al., 2001). These domains are defined based on either the β(M283) or β(M286) residues at the β+α interface in the transmembrane domain (TMD) and the β(Y143) residue close to the β-surface in the junction between the extracellular domain (ECD) and TMD (Figure 4B) (Shin et al., 2018; Borghese et al., 2021). Although it has been found that the β subunits are important for SPD-mediated regulation of GABA_A_Rs, subtypes containing β3 subunits are the primary targets (Jurd et al., 2003; Eaton et al., 2015; Kreuzer et al., 2020). In mutant β3(N265M) mice, the action of propofol via β3-containing GABA_A_Rs was severely restricted. It has also been speculated that the propofol binding sites are preferably located at the αβ+/α−γ interface and the α+/β− or α+/γ− TMD interfaces in the α1β3γ2 isoform (Bali and Akabas, 2004; Maldifassi et al., 2016). In addition, photolabeling of β3 homomeric and α1β3 receptors also suggested that propofol is in contact with β3H267 (Yip et al., 2013). However, the results of a mutational analysis do not support the idea that β3H267 is a propofol binding site (Stern and Forman, 2016; Feng and Forman, 2018).

### 3.3 Bisphenols

The first study reporting the action of BPA on GABA_A_Rs was conducted on recombinant α1β1 isoforms expressed in *Xenopus* oocytes as shown in Figure 4) (Aoshima et al., 2001). Depending on its concentration, BPA has dual action on GABA_A_Rs; ≤100 μM BPA causes an increase in GABA-mediated Cl^−^ currents while >100 μM BPA causes a decrease. This action was found to be induced by the direct binding of BPA to GABA_A_Rs in an estrogen-independent manner (Figure 4A). Other authors investigated the action of BPA on GABA-induced Cl^−^ currents (I_GABA_) in isolated rat CA3 pyramidal neurons (Choi et al., 2007). In those studies, <10 μM BPA potentiated the GABA-mediated I_GABA_ peak, while diazepam (or ethanol) led to a great reduction in the BPA-mediated activation of I_GABA_. However, >30 μM BPA caused suppression of the peak I_GABA_ mediated by GABA and accelerated the desensitization state. BPA (>30 μM) inhibited the steady-state GABA-mediated I_GABA_) in a noncompetitive manner. Perinatal administration of BPA at low doses produced an inhibition of GABA_A_R-mediated Cl^−^ currents in neurons of the amygdala, leading to abnormal cortical-amygdala synaptic transmission and inducing neurobehavioral alterations (Zhou et al., 2011); however, the long-term behavioral effects of developmental BPA exposure can be reversed (Soriano et al., 2016).

According to data from various studies, BPA-mediated memory/learning disorders may be caused by altering GABA_A_R α1 subunit expression and distribution in the CA1 region of the hippocampus and prefrontal cortex. Specifically, BPA administration caused a considerable reduction in the density of GABA_A_R α1 subunits in the prefrontal cortex and hippocampus (Taherianfard and Taci, 2015). The distribution of GABA_A_Rs was denser in BPA-exposed rats that were subject to learning than in non-learning rats. Recent studies have reported sex-specific effects of long-term BPA administration on the level of α2 subunit expression in the hippocampus with increased levels in females and decreased levels in males (Xu et al., 2015). These data concerning behavioral alterations prompted the authors to suggest that long-term exposure to BPA affects anxiety- and depression-like behaviors in adult mice, which are mediated by changes in the expression levels of the GABA_A_R α2 subunit in the hippocampus.

The binding sites of BP on the GABA_A_R have not yet been identified, but they may be close to the sites for neurosteroid binding, which are distinct from sites at which other phenols bind (Figure 4B). Endogenous steroids display GABA_A_R-mediated neuroactive effects, including anesthesia, analgesia, and sedation. Although the exact location of the neurosteroid-binding sites has yet to be determined, many residues in the TMDs (αS240, αQ241, αN407, αY410, αT236, and βY284) have been shown to affect neurosteroid activity (Li et al., 2009). The modulatory and activation sites of neurosteroids are located at the TMDs of the α subunit and β+/α–interfaces, respectively (Miller et al., 2017). In particular, photolabeling of β3 homomeric receptors identified F301 in β3-M3 as a possible neurosteroid binding site (Chen et al., 2012).

### 3.4 Polyphenols

Complex polyphenols, such as flavonoids, terpenoids, and polyacetylenic alcohols, affect the GABA system (Hanrahan et al., 2011; Ríos et al., 2022). Such compounds can manifest as agonists (such as thymol), inhibitors (such as picrotoxinin, bilobalide, or ginkgolide), or allosteric modulators of GABA_A_R activity (Figure 3). Moreover, glycosylation reduces the binding of natural phenols to GABA_A_Rs (Wang et al., 2002). Flavonoids can also interact with flumazenil-sensitive or -insensitive GABA_A_Rs (Hanrahan et al., 2015). Several studies have shown that some isoflavones (such as genistein or tyrphostin) are protein tyrosine kinase (PTK) inhibitors that can directly block GABA_A_R function or act in a kinase-dependent manner to block receptor function (Dunne et al., 1998; Huang and Hsu, 1999). Earlier studies have shown that GABA_A_R-mediated Cl^−^ currents are directly depressed by genistein or tyrphostin in neuronal and recombinant GABA_A_Rs (Moss et al., 1995; Jassar et al., 1997; Wan et al., 1997; Castel et al., 2000). The flavone, hispidulin, was found to potently activate the α6β2γ2 GABA_A_R isoform and led to a reduction in susceptibility to seizures (Kavvadias et al., 2004). These data indicate that flavonoids can be considered GABAergic agents (Figure 4A). Although the role of flavonoids in GABA_A_R regulation has received attention (Hanrahan et al., 2011; Ríos et al., 2022), the molecular mechanisms underlying their direct actions on GABA_A_Rs remain poorly understood.

Many flavonoids (including isoflavones and flavones) possess modulatory actions on the benzodiazepine-binding site of GABA_A_Rs (Hanrahan et al., 2015). However, the compounds within this family show potential actions at more than one additional binding site on GABA_A_Rs (Figure 4B). Notably, benzodiazepine-sensitive GABA_A_R subtypes comprise two α and two β subunits in addition to one γ subunit (Fritschy, 2008). GABA_A_Rs containing α4, α6, γ2, and, to a lesser extent, δ, subunits, can potently bind many benzodiazepine ligands (Olsen and Sieghart, 2009; Ghit et al., 2021). Mutations that convert histidine to arginine (α1H101R, α2H101R, α3H126R, and α5H105R) of the β2γ2 subtype of GABA_A_Rs eliminated diazepam activity, whereas reverse mutations elicited diazepam responses (Benson et al., 1998).

## 4 GABA_A_Rs and neuropsychiatric disorders

Although data concerning the role of GABAergic receptors in different areas (including cortex, hippocampus, hypothalamus, amygdala or spinal cord) of the brain are limited, evidence that the same GABA_A_R subtypes which are expressed in various neuronal populations can modulate the excitability and neuronal synchronization. Accumulated data in the literature indicate that disturbance of GABA_A_ergic signaling is a root cause for the appearance of neuropsychiatric disorders (Chiapponi et al., 2016; Van Nuland et al., 2020). To confirm the interaction of phenols with GABA_A_Rs in the development of neurobiological consequences, we considered the role of these receptors in the manifestation of neurological and psychiatric diseases. In this chapter, we investigated correlations between deficiencies in specific GABA_A_R subunits and the occurrence of NPDs.

### 4.1 Tremor

Although it is believed that bradykinesia results from a reduction in dopaminergic neurons (Calon et al., 2003), research done over the last two decades revealed that the major pathophysiological paradigm underlying tremors is the GABA_A_R hypothesis (Boecker et al., 2010; Paris-Robidas et al., 2012; van Nuland et al., 2020). In addition, the reciprocity between dopamine and GABA in the basal ganglia has been recognized (Hoerbelt et al., 2015). Recently, it was shown that dopamine without agonists can directly regulate recombinant GABA_A_Rs by interacting with the β3 subunit. The etiology of essential tremors involves the abnormal firing of Purkinje cells, which receive excitatory inputs from granule cells in the cerebellum (Nietz et al., 2020). A previous study showed that the Purkinje cell-specific knockout of the GABA_A_R α1 subunit eliminated all GABA_A_R-mediated inhibition in Purkinje cells while retaining the GABA_A_R-mediated inhibition of intact cerebellar molecular layer interneurons. The selective depletion of the GABA_A_R α1 subunit from Purkinje cells did not induce deficiencies in the accelerating rotarod test or decreased survival rates. However, an essential tremor-like phenotype has been observed in animals with a global knockout of the GABA_A_R α1 subunit (Nietz et al., 2020). This finding is similar to the essential tremors observed in patients. In contrast, results from recent studies provide important new clues into tremor suppression mechanisms initiated by the enhancement of GABA-driven inhibition in pathways controlled by the α2/3 GABA_A_R subunits but not the α1 subunit (Kosmowska et al., 2023). In addition, GABA_A_R α6 subunit-selective drugs were found to cause a substantial reduction in tremors and restoration of physical wellbeing in a mouse model (Huang et al., 2021).

### 4.2 Epileptic seizures

The GABA_A_R β3 subunit is highly expressed in immature and adult brains, specifically in circuits involved in seizure generation and epileptic seizures (Hörtnagl et al., 2013). Moreover, changes in the physiological and biochemical properties of β3-containing GABA_A_R assemblies in the brains of patients with epilepsy (Menzikov et al., 2021). Several studies have reported that *GABRB3* mutations with juvenile myoclonic epilepsy, childhood absence epilepsy, and/or other syndromes and that reduced GABA_A_R function causes an E: I imbalance (Gurba et al., 2012; Janve et al., 2016). Animal models of epilepsy have demonstrated obvious alterations in the expression and rearrangement of GABA_A_R subunits in the hippocampus and the para-hippocampal areas, including downregulation of the α5 and δ subunits and upregulation of the α4 subunit (Sperk et al., 2021). Findings addressing the increased expression of the α4 subunit in patients with temporal lobe epilepsy are similar to those observed in rodent models (Schipper et al., 2016).

### 4.3 ADHD

Although the etiology and pathophysiology of ADHD remain unclear, some data support the interaction between genetic and environmental factors (Wang et al., 2012; Sun et al., 2018). In particular, Kwon and coauthors have shown an association between *GABAB3* gene polymorphisms and ADHD in children (Kwon et al., 2017). Rodents with a deficiency in the β3 subunit exhibit thalamic disinhibition, seizures associated with learning/memory deficits, hyperactivity, poor motor skills in repetitive tasks, and a disturbed rest–activity cycle (DeLorey et al., 1998).

### 4.4 Anxiety/depression

Accumulating data suggest that the major cause of depressive disorder is GABAergic dysfunction (Luscher et al., 2011; Nuss, 2015). Specifically, it was previously established that cortical GABA_A_Rs decreased in patients with depressive pathologies (Fogaça and Duman, 2019). In addition, abundant evidence that the expression of various GABA_A_R subunit transcripts is altered in depression in animal models has been reported. For example, partial GABA_A_R deficits in mice (heterozygous γ2 or α2 subunits) induced depression-like behavior (Möhler, 2012) and found that *GABRA1* gene expression is subject to epigenetic control (Luscher et al., 2011). In addition, the behavioral deficit was restored by chronic antidepressant treatment in γ2 mice (Luscher et al., 2011). Analysis of the frontopolar cortex of suicide victims who had suffered from various forms of depressive disorders revealed reductions in the abundance of α1, α3, α4, and δ subunit mRNAs (Oquendo et al., 2014). A comparison of the brains of suicide victims with or without depression showed elevated mRNA expression levels of the α5, γ2, and δ subunits in the dorsolateral prefrontal cortex (Merali et al., 2004). However, some studies suggest that variations in the gamma subunits, GABRA2, GABRA3, GABRA6, and GABRG2, do not play a key role in the susceptibility to anxiety spectrum disorders (Pham et al., 2009). Recent anatomical and electrophysiological data indicate that α6-containing GABA_A_Rs in cerebellar granule cells can be specific targets for treating NPDs as described in several studies (Sieghart et al., 2022).

### 4.5 Schizophrenia

A deficit in GABA_A_R signaling is the current hypothesis underlying the manifestation of schizophrenia (Chiapponi et al., 2016). A decrease in the mRNA expression of the GABA_A_R α1, γ2, α4, α5, and δ subunits in the dorsolateral prefrontal cortex (DLPFC) of patients with schizophrenia was found (Gill and Grace, 2014; Mueller et al., 2015; Marques et al., 2021). Changes in the expression of the GABA_A_R α2, β1, and ε subunits in the lateral cerebellum were found in patients with schizophrenia, depression, and bipolar disorder (Fatemi et al., 2013; Fatemi et al., 2017). Meanwhile, several reports about an increase in the mRNA expression of the GABA_A_R α1 and α5 subunits and no change in the expression of the α4 receptor subunit in schizophrenic brains of humans (Maldonado-Avilés et al., 2009; de Jonge et al., 2017). Several studies on schizophrenic brains showed a decrease in high-mannose N-glycan levels in GABA-associated proteins specific to different GABA_A_R subunits (α1, α4, and β1–3), increased levels of high-mannose N-glycans in the β1 subunit, decreased levels of high-mannose N-glycans on the α1 subunit, and alterations in the total N-glycan content of the β2 subunits (Mueller et al., 2014; Williams et al., 2020).

### 4.6 Memory/learning

The GABA_A_R α5 subunit preferably localizes in the hippocampus of the mature brain. It is related to learning and memory and was discovered through the targeted disruption of the α5 gene in mice (Collinson et al., 2002) in which it was found that α5^−/−^ mice demonstrated elevated spatial learning performance. In contrast, no changes in performance were observed in non-hippocampal-dependent learning and anxiety tasks. In addition, α5^−/−^ mice showed a decrease in the amplitude of inhibitory postsynaptic currents (IPSCs) and an increase in the paired-pulse facilitation of field excitatory post-synaptic potentials (EPSP) in the CA1 region of the hippocampus. Wiltgen et al. showed an increase in the expression of the α1 subunit in the lateral nucleus of the amygdala (Wiltgen et al., 2009). Administering an α1 subunit antagonist into the lateral amygdala caused a selective impairment in auditory learning. Mice with a selective knockout of the α1 subunit in excitatory cells did not exhibit enhanced learning.

In the hippocampus, GABAergic tonic currents are tightly associated with memory and play an essential role in cognition. In the extra-synapses, the distribution density of α5-containing GABA_A_R subtypes is relatively high in the hippocampus, which can be considered an amnesia-like mechanism during anesthesia (Zhao et al., 2019). Sedation refers to a decrease in the arousal level as indicated by longer response times, decreased motor activity, and slurred speech. In animal models, sedation is characterized by reduced motor activity and arousal (Rudolph and Antkowiak, 2004).

### 4.7 Autism

Several studies concluded that an impairment in the E: I balance is the main reason for autism development, which was confirmed in several mouse models of autism (Paine et al., 2020). Mendez and coauthors conducted a positron-emission tomography (PET) imaging study using the radioactive ligand [^11^C]-Ro15–4513 VT to trace levels of the GABA_A_R α5 subunit in autism (Mendez et al., 2013). Their results showed a reduction in the expression of the GABA_A_R α5 subunit in two limbic areas of the brains (amygdala and nucleus accumbens) of autistic patients. In contrast, a recent study showed that disturbance in the GABAergic system in autism mouse models and patients with autism was not associated with alterations in the number of GABA_A_Rs between healthy and diseased individuals (Horder et al., 2018), and based on a meta-analysis, which also failed to show a link between changes in the GABA_A_R subunits (β3, α5, and α3) and autism in children (Mahdavi et al., 2018).

## 5 Molecular mechanisms underlying the action of phenols on GABA_A_Rs

The capability of phenols to interact with different target structures and cause a broad range of effects can be attributed largely to their amphiphilic character. Indeed, an oxygen atom normally forms δ bonds with other atoms (as an example, with hydrogen atoms in H_2_O molecule). When one hydrogen atom is missing in a water molecule, the formation of the -OH group, which is responsible for various physical and chemical features of various compounds, including phenols, is prone to electrophilic substitution reactions due to its rich electron density. In particular, these reactions are initiated by reactions of electron-deficient groups with the negatively charged oxygen atom or by reaction of electron-rich groups to the positively charged atoms (C or H bonds) whereas the availability of the hydrophobic planar benzene ring in phenolic molecules is responsible for π-binding (π-stacking) and other non-covalent interactions. Such structural peculiarities allow phenols to induce positive and/or negative effects on various target proteins, including enzymes, receptors, and lipid structures, which subsequently modulates their properties (Tungmunnithum et al., 2018; Lin et al., 2021). Thus, the action of phenols on GABA_A_R function could result from their specific binding with their target subunits, nonspecific interactions with surrounding membrane lipids, or perturbation of the cytoskeleton in the receptor environment (Figure 4C).

### 5.1 Via hydrogen-bond formation

Hydrogen bonds are important in potentiating diverse cellular functions, including protein–ligand interactions (Chen et al., 2016). Some authors demonstrated that tyrosine H-bonds largely contribute to protein stability and are considered the strongest conventional H-bonds (Pace et al., 2001; Scheiner et al., 2002). Compounds containing -OH groups in their structure interact with receptors via H-bond formation (Starovoytov et al., 2014). H-bonds usually occur between the phenolic OH-group and the protein–peptide bonds; the strongest of all is the conventional H-bonds, namely, OH‧‧O and OH‧‧N (Scheiner et al., 2002). SPs and SPDs have been shown to interact with GABA_A_Rs partly via the formation of H-bonds between their -OH groups and various amino acid residues (Figure 4C). Specifically, H-bonds form between the −OH groups of phenol compounds and amino acid residues within the binding cavity of the GABA_A_R β3 subunit (Krasowski et al., 2001). In addition, a structure analysis suggests a direct interaction between propofol and the phenol residue of tyrosine in the receptor channel (Leon et al., 2012). The strengths of H-bonds at interaction with aromatic amino acids have followed this trend–Trp > His > Tyr ∼ Phe. In other studies, the formation of H-bonds between phenols and amino acid residues was used as an introductory model for biological systems because of their structural similarities to tyrosine, a para-substituted phenol (Fedor and Toda, 2014). However, the molecular mechanism and the intensity by which H-bonds regulate molecular interactions of phenol with GABA_A_Rs remain unclear because the H-bonding continuously competes with bulk water (Utas et al., 2006).

### 5.2 Via water displacement

Some authors suggest that phenol might modulate rat recombinant α_1_β_2_γ_2s_ GABA_A_Rs by replacing H_2_O from one or more low-affinity amphipathic binding sites, resulting in a conformational rearrangement that increases anion conductance as shown in Figure 4C (Brosnan and Pham, 2016; Brosnan and Pham, 2018). It was previously suggested that the presence of amphipathic receptor sites normally occupied by H_2_O molecules is associated with dissociation constants inversely related to the cut-off solubility value of phenols, which is between 0.10 and 0.46 mM. Weakly soluble phenol compounds cannot reach concentrations sufficient to compete with H_2_O for binding-site access and, therefore, fail to modulate GABA_A_Rs (Brosnan and Pham, 2018).

### 5.3 Via membrane lipid modification

Various phenols can modify the properties of membranes, including their fluidity, thickness, or lateral structure, with several conformational changes in membrane proteins (Tsuchiya and Mizogami, 2014). Several studies used various phenols, model lipids, and measurement methods to collect detailed information on phenol–lipid interactions (Figure 4C). In particular, it was shown that various phenol compounds at concentrations of 1–10 μM interacted with the model or neuronal membranes to raise their fluidity in the order of potency: propofol > thymol > isothymol > guaiacol > phenol > eugenol, which is consistent with the order of their activity (Tsuchiya and Mizogami, 2020). In addition, these chemicals caused a reduction in membrane lipid peroxidation at potencies correlating with their membrane activities. Miguel et al. (2019) used an equilibrium molecular dynamics simulation approach and showed that applying SPDs in bilayers affected lipid acyl chains in carbons near the interface, but their influence is negligible at the center of the bilayer. Some authors who use nuclear magnetic resonance (^1^H-NMR) spectroscopy showed that GABAergic phenol derivatives can be incorporated into phospholipid vesicles, specifically within the region between polar groups (choline molecules), glycerol, and the first atoms of the acyl chains (Reiner et al., 2013). Furthermore, inserting these chemicals into membranes leads to a reduction in the repulsion between the phospholipid head groups and a reduction in the overall mobility of the hydrocarbon chains. Natural polyphenols can also interact with the membrane and penetrate lipid bilayers depending on the structure, concentration, and composition of the membrane lipids (Bonarska-Kujawa et al., 2011; Karonen, 2022).

### 5.4 Via cytoskeletal modification

It is known that pLGICs interact with signaling and cytoskeletal proteins (Sheng and Pak, 2000; Björnström and Eintrei, 2003). Phenol compounds can induce reorganization of the actin cytoskeleton in neurons into ring structures (Figure 4C). For example, the interaction between propofol and actin triggers a dose-dependent internalization of GABA_A_R β2 subunits (Oscarsson et al., 2007); such an increase in internal GABA_A_ β2 subunit content is closely related to actin polymerization.

Using fluorescence-labeled actin in cultured rat neurons, several authors evaluated the percentage of actin rings induced by propofol or GABA in combination with Rho, Rho kinase (ROCK), phosphoinositide 3-kinase (PI3K), or protein tyrosine kinase (PTK) inhibitors (Björnström et al., 2008). In contrast to GABA, propofol induces transcellular actin ring organization that is dependent on the influx of extracellular calcium, which can be blocked by ROCK, PI3-kinase, or tyrosine kinase inhibitors. Thus, propofol utilizes Rho/ROCK to facilitate actin translocation from the cytoskeleton to the plasma membrane, while actin ring organization depends on an interaction site close to the agonist site on GABA_A_R. GABA did not mediate actin ring formation, indicating that this effect was propofol-specific.

### 5.5 Via kinase regulation

GABA_A_R phosphorylation governs numerous processes, including channel activity regulation, control of receptor trafficking, effects on receptor-interacting proteins, and altering sensitivity to various drugs (Balduzzi et al., 2002). Cyclic-AMP-, Ca^2+^/phospholipid-, Ca^2+^/calmodulin-, and cGMP-dependent kinases catalyze the transfer of γ-phosphate from ATP to a serine or threonine residue, which typically causes a reduction in GABA_A_R function (Nakamura et al., 2015). In recent decades, studies have demonstrated the importance of PTKs in regulating the properties of GABA_A_Rs (Moss et al., 1995). Tyrosine residue phosphorylation by PTKs (Src family) causes an increase in receptor activity (Jassar et al., 1997). The tyrosine residues of the β3 subunit (Tyr365 and Tyr367) and serine residue of the γ2 subunit (Ser343) are substrates of PTKs as shown in Figure 3C (Wan et al., 1997). In cultured frog pituitary melanotrophs, extracellular application of the PTK blocker, genistein, can cause a bell-shaped modification of the whole-cell GABA_A_R-mediated Cl^−^ currents (Castel et al., 2000). In particular, low concentrations of genistein (<0.1 μM) induced receptor activation, whereas high concentrations (>50 μM) caused a reversible reduction in the GABA_A_R-mediated Cl^−^ current. In addition, administration of recombinant PTK pp60c-src (inside-out configuration) inhibited GABA_A_R-mediated Cl^−^ currents; this effect was reversed by genistein (Figures 4A–C). Immunoblotting revealed that genistein markedly inhibited the tyrosine phosphorylation of GABA_A_R β2/β3 subunits. In addition, propofol caused an increase in the intracellular calcium levels ([Ca^2+^]_i_) of primary neurons cultured from newborn rats when exposed to neurons cultured in a Ca^2+^-free medium (Björnström et al., 2002). This increase in [Ca^2+^]_i_ declined when the cells were preincubated with the PTK blocker, herbimycin A. Propofol treatment caused an increase in tyrosine phosphorylation of the GABA_A_R β subunits. Thus, PTKs participate in these propofol-mediated biological effects by inducing calcium release from intracellular stores and modulating the GABA_A_R β subunits (Björnström et al., 2002). In addition, polyphenols isolated from the Chinese mangrove plant can inhibit GABA_A_Rs via individual kinases (IC_50_ = 2–4 μg) as described in several studies (Shi et al., 2010).

### 5.6 Via modification of receptor desensitization

**FIGURE 5:**
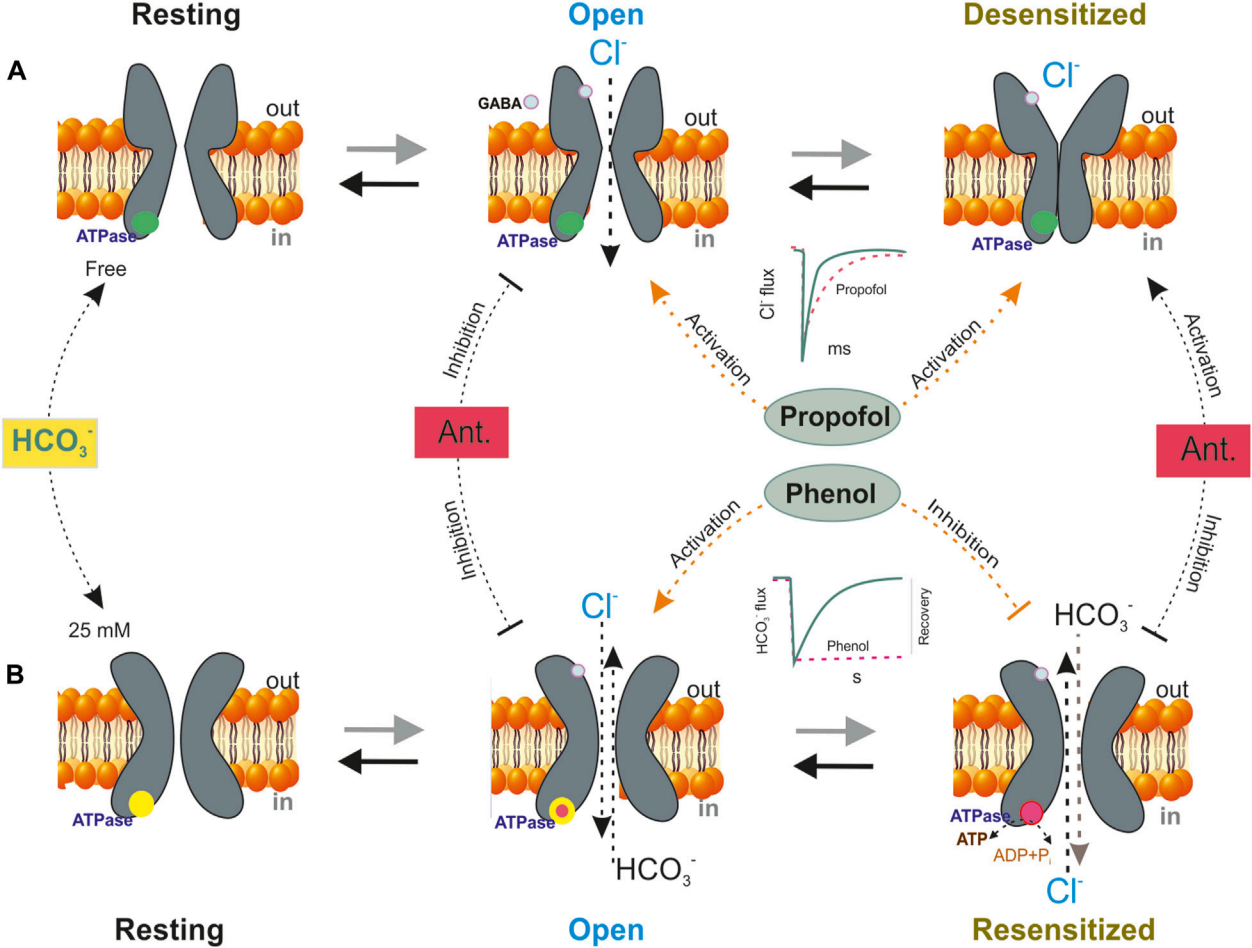
A schematic model showing four basic conformation states that depict the GABA_A_R function–a resting, an open, a desensitization or resensitization states. **(A)** In an HCO_3_^−^ free medium, GABA (green circles) exposure shifts the equilibrium from resting to open state and on repeat (or prolongation) mediator application is observed the GABA_A_R desensitization. Propofol influences the GABA-mediated response by stabilizing different states, including resting, desensitized conformations and controlling their transitions. The blue circles are the ATPase in the none active form. The most noticeable effect of propofol on the responses to short exposures to mediators is that it prolongs deactivation. **(B)** In the presence of physiological concentration of HCO_3_^−^ (25 mM), GABA changes the conformation of channel and shifted the equilibrium from resting state to open state. Bicarbonate determines the phenol action on the GABA_A_R. Phenol activates the open state and inhibits the resensitization state of the receptor − ([Cl^−^]_i_/[HCO_3_^−^]_i_ recovery. The GABA_A_R-coupled ATPase only possesses low sensitivity (>1 mM) to phenol effect if it is activated only Cl^−^ or HCO_3_^−^, whereas the Cl^−^, HCO_3_^−^ ATPase is inhibited by low concentrations of phenols (<1 mM). It was shown that the transition from the desensitization state to resensitization is coupled with a fall in [ATP]i, which is carried out via ATPase performance. The red circles are the ATPase in the active form. Antagonists (Ant.–picrotoxin or bicuculline) inhibit the open state but activate the desensitization state.

GABA_A_Rs quickly open their transmembrane pores upon neuromodulator binding thus enabling anions to flow passively into neurons via the plasma membrane (Ghit et al., 2021). However, GABA_A_Rs undergo desensitization following activation, which provides incremental entry into a long-term closed state refractory to excessive or repeated activation (Figure 5) (Field et al., 2021). The desensitization process causes a decrease in Cl^−^ currents and facilitates GABA entry into a bound state with GABA_A_Rs (Bai et al., 1999). Some studies addressing desensitization have focused on changes in the conformation of GABA_A_Rs following significant activation by activators or allosteric modulators (Kang et al., 2020). In addition, functional and structural data support a “dual-gate” model in which the TMD of pLGICs contains both an activation gate, located at the top of the channel, and a deactivation gate, located at the intracellular end of the channel as shown in Figure 5 (Gielen and Corringer, 2018; Kaczor et al., 2021). Recent structural studies have shown conformational changes between the mediator-bound open state and the desensitized state that occurs at the “internal face” of the receptor (Rovšnik et al., 2021). Such alterations also include phosphorylation of GABA_A_R subunits by protein kinases, their expression, clustering, and pharmacology.

Although sufficient structural and pharmacological data are present, our understanding of the effects of different phenols on receptor desensitization is formed mainly by studies conducted using propofol (Borghese et al., 2021) or neurosteroides (Miller et al., 2017). The most noticeable effect of propofol on the responses to short exposures to mediators is that it causes prolongation of deactivation. Specifically, an earlier study noted that propofol prolongs desensitization as evidenced by changes over the time course of the GABA responses (Orser et al., 1994). In addition, propofol influences the GABA-mediated response by stabilizing different states, including the resting and desensitized conformations in addition to controlling the transition between these states (Figure 5). Such channel activation and changes of desensitization may result from propofol binding near the GABA recognition site. Several structural studies have shown at least five certain receptor states, namely, three nonconducting states (resting, diazepam-bound, and potentiating propofol-bound) and two conducting-desensitized states (activating propofol-bound and mediator-bound states) as described in several studies (Williams and Akabas, 2002; Shin et al., 2018).

### 5.7 Via modification of receptor resensitization

Regulating GABA_A_R desensitization is an essential process that is controlled at the signaling pathway and receptor levels; however, modulating receptor function at the level of the energy processes plays a key role in understanding withdrawal in neurological and psychiatric disorders (Rezin et al., 2009). Mitochondrial dysfunction is a key factor in epilepsy, Parkinson’s disorder, schizophrenia, and other synaptic and extra-synaptic pathologies (De Simone et al., 2023). However, the relationship between GABA_A_R activity, impaired energy balance, and manifestations of NPDs remains poorly understood.

Early research has shown that exposure to SPs (such as catechol and pyrogallol) induces convulsive activity accompanied by a rapid decline in neuronal ATP levels ([ATP]_i_) in the brains of mice (Angel et al., 1969; Angel and Rogers, 1972). Other studies showed that neuronal [ATP]_i_ led to a decrease in energy production (P_i_/min/g), which is elevated abruptly during seizures and recovered after phenobarbital treatment (Stanley and Mantz, 1971; Winn, 1980; Walton et al., 1998). However, these studies reported that the reduction in [ATP]_i_ was partly due to mitochondrial dysfunction that occurred during the seizure state; the main reason behind the reduction in [ATP]_i_ during neurological disorders is still elusive (Walton et al., 1998). Furthermore, many reports have demonstrated that even due to the effects of various ligands (including GABA), the reduction in [ATP]_i_ is the main reason underlying the reduction in GABA_A_R functional activity, which ultimately manifests as neuronal excitation (Sallard et al., 2021). However, the molecular events determining the decline in ATP levels during the GABA_A_R run-down phenomenon and desensitization state remain unknown (Figure 5).

Many literature reports show that GABA_A_R desensitization is tightly associated with its slow deactivation, namely, so-called process of resensitization (Hinkle and Macdonald, 2003; Kang et al., 2020). Although some kinases play a role in the desensitization and retardation of GABA_A_R deactivation, the main players involved in the resensitization process have not been fully established. Recently, it was reported that the β3 and β1 subunits possess a phenol-regulated ATPase, which is localized in the ICD (Menzikov, 2018; Menzikov et al., 2021) and that resensitization by the enzyme facilitates the replenishment of the neuronal concentrations of chloride ([Cl^−^]_i_) and bicarbonate ([HCO_3_^−^]_i_) after GABA_A_R activity and desensitization (Menzikov et al., 2021; Menzikov et al., 2022). A recent finding showed that the Cl^−^ ATPase activities belonging to the β1 and the β3 subunits have Cl^−^, HCO_3_^−^ATPase activity, which determines differences in their sensitivity to phenol (Menzikov et al., 2023). Furthermore, this enzyme is within the scope of GABA_A_R performance as Cl^−^ATPase during the first week of postnatal rodent development and then as a Cl^−^, HCO_3_^−^ATPase. The GABA_A_R-coupled ATPase only possesses low sensitivity (>1 mM) to the phenol effect if it is activated only by Cl^−^ or HCO_3_^−^, whereas the Cl^−^, HCO_3_^−^ ATPase is inhibited by low concentrations of phenols (<300 μM). It was shown that the transition from the desensitization state to resensitization is coupled with a fall in [ATP]i, which is carried out via ATPase performance (Figure 5). The critical role of ATPase during the resensitization process and the resulting conformational changes in GABA_A_Rs were confirmed using a thiol alkylating agent and a mutant receptor (Menzikov et al., 2022). Phenol modulates the formation of a phosphoprotein (high-energy phosphate bond) and the rate of ATP-consuming Cl^−^ transport by the ATPase (Menzikov and Morozov, 2019). Moreover, it was established that phenol interacts with the GABA_A_R β3 subunit by binding with the ATP-hydrolyzing site of ICD a finding that directly confirms the interaction of phenol with GABA_A_Rs.

## 6 Phenols and ionic plasticity

Neuronal/synaptic plasticity concerns structural and functional alterations that take place in different parts of the brain over a range of timescales and adapt their function in response to specific stimuli. Ionic plasticity is directly associated with the modulation of the functional expression and properties of ionic channels. Neuronal chloride ([Cl^−^]_i_) and bicarbonate ([HCO_3_^−^]_i_) ion concentrations are pivotal parameters that control the E: I balance, and their effects depend on specialization and the level of neuronal development. Ionic plasticity of GABAergic signaling refers to the modulation of neuronal functions via changes in the driving force (DF_GABA_) of the underlying anionic currents via short- and long-term mechanisms that can lead to significant changes (E_GABA_) (Raimondo et al., 2017; Sallard et al., 2021). Specifically, GABA_A_R dynamics is the result of the short-term changes in the neuronal Cl^−^ and HCO_3_^−^ gradients that control the nature and strength of GABA_A_R-mediated currents (E_GABA_), whereas activity-dependent changes in the trafficking, kinetics, and function of [Cl^−^]_i_/[HCO_3_^−^]_i_ regulating systems (cation-chloride cotransporters, carbonic anhydrases, and Na^+^, K^+^ATPase) can result in long-term shifts in the DF_GABA_. For example, in mature neurons, the [Cl^−^]_i_ is low, and GABA_A_R activation triggers Cl^−^ influx into neurons, which results in the hyperpolarization of the TMP. Under certain conditions (such as the massive action of ligands or spinal cord lesions), the [Cl^−^]_i_ can reach high values, close to the [Cl^−^]_i_ in immature neurons (Fritschy, 2008). In this state, GABA can shift the TMP from hyperpolarization toward depolarization and even excitation, which depends not only on Cl^−^ influx but also on HCO_3_^−^ efflux via the channel (Fujiwara-Tsukamoto et al., 2003; Kaila et al., 2014). Several reports showed that changing the [Cl^−^]_i_ is essential for desensitization (Ghit et al., 2021), whereas changing the [HCO_3_^−^]_i_ is essential for receptor resensitization (Menzikov et al., 2022).

**FIGURE 6:**
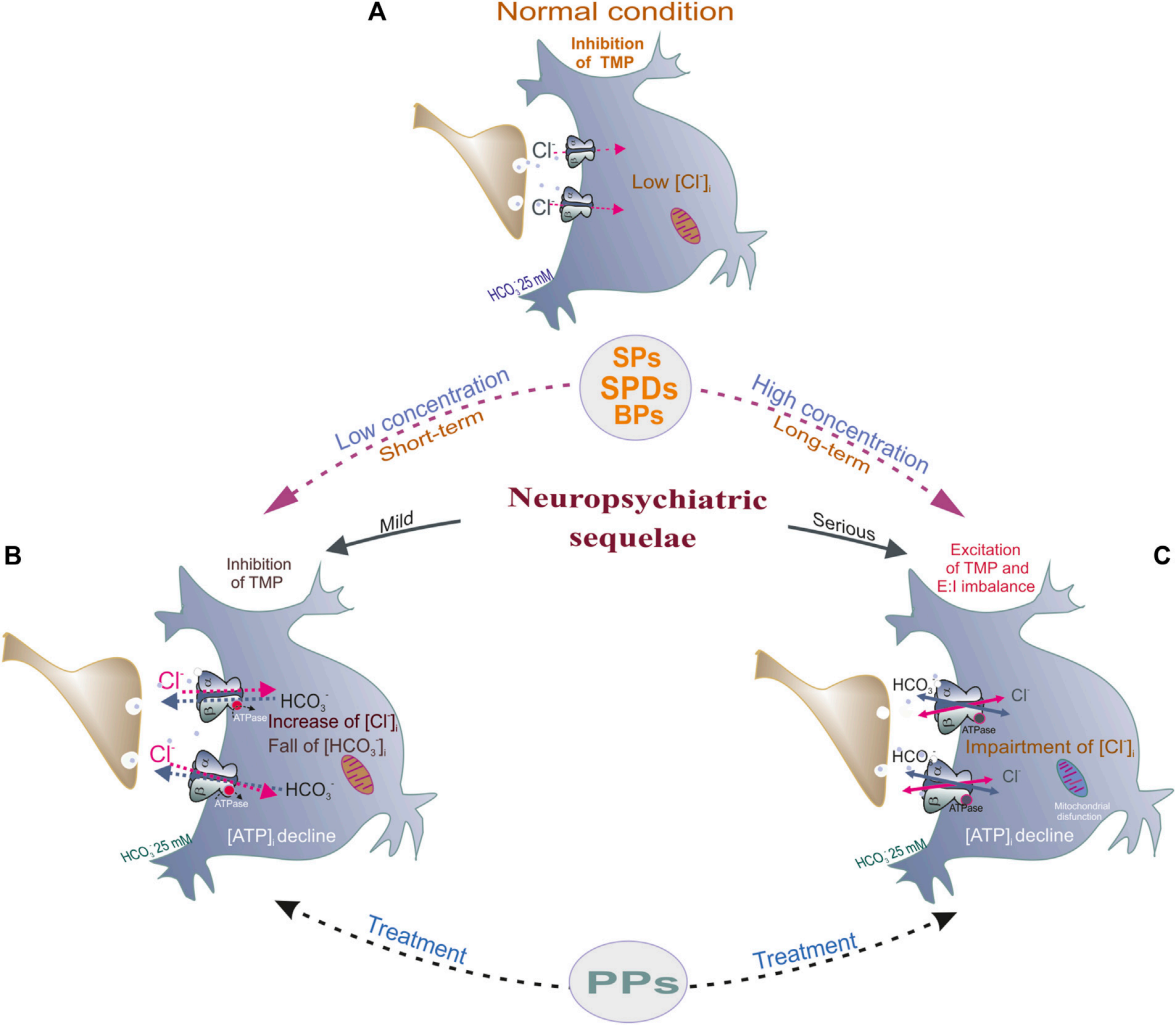
Potential mechanism underlying phenol-mediated changes of E:I balance by modulation of GABA_A_Rs function. **(A)** In normal condition, in mature neurons, the [Cl^−^]_i_ is low, and GABA_A_R activation by GABA (green circles) triggers a fast Cl^−^ influx and subsequent hyperpolarization/inhibition of the TMP. **(B)** Low concentration (or short-term of phenols (SPs, SPDs or BPs) exposure induced an essential increase of Cl^−^ influx and HCO_3_^−^ outflux via receptor pore and as a result, the increase of hyperpolarization of the TMP. Here, phenol compounds increase the GABA_A_R-coupled Cl^−^, HCO_3_^−^ATPase activity, the decline of [ATP]_i_, and what accompanies by mild neuropsychiatric pathologies (head twitching, tremor). **(C)** High concentration (or long-term) of phenols application induced impaired of synchronicity of multidirectional Cl^−^ and HCO_3_^−^ fluxes via receptor pore, ATPase inhibition and as a result, the depolarization of TMP, E:I imbalance (collapse). These pathologies are characterized by the decline of [ATP]_i_, mitochondrial dysfunction and accompanies by serious neuropsychiatric manifestation.

Interneuronal GABA_A_ receptors inside and outside of synapses are required for normal brain function, including plasticity, learning/memory, and neuronal networks. Phenols interact with GABA_A_R subunits (preferably β subunits) and act as activators (such as SPs and SPDs) or inhibitors (PPs) depending on the complexity of their structure (Figure 3). A comparative analysis of various chemicals belonging to various phenolic groups demonstrated similarities and differences in their capabilities to regulate GABA_A_Rs and subsequently cause neuropsychiatric disorders. A certain pattern can be observed between the structure of phenolic substances and their effects on receptors (activation or inhibition); the more complex the structure of the substance, the more likely they are to elicit inhibitory effects (Figure 3). Other literature studies also demonstrate that the effect of many phenols (including natural compounds) on the CNS exhibits a concentration dependence; at low concentrations, it activates, and at high concentrations, it inhibits receptor function (Borghese et al., 2021). In addition, *in vivo* and *in vitro* studies have shown that administering low phenol concentrations caused an increase in GABA_A_R-coupled ATPase function and induced mild manifestations of the disease in animals, whereas the effect of high concentrations of phenols on receptor activity may represent another side of their influence on GABA_A_Rs; higher phenol concentrations inhibit ATPase activity but mediate the development of severe disorders. Thus, phenols activate the passive permeability of anions via GABA_A_R channels into neurons (Brosnan and Pham, 2018; Menzikov and Morozov, 2019) but inhibit the GABA_A_R-coupled ATPase activity, which recovers anion homeostasis in neurons that can be associated not only with different sites of binding but and modification of non-covalent interactions (firstly, H-bonds) as shown in Figure 6. Moreover, non-covalent interactions between phenols and amino acid residues are quite labile and especially dependent on the pH value or ionic strength (Schefer et al., 2021). Such a double role of phenols in the modulation of the GABA_A_R dynamic is prone to disrupt the short- and long-term alterations of ionic plasticity and, as a result, impact neuronal network activity and neuropsychiatric sequelae. As noted in previous chapters, phenol compounds with varying potency can impair GABA_A_R desensitization/resensitization and cause a collapse of neuronal Cl^−^ and HCO_3_^−^ gradients that ultimately cause neurobiological sequelae (Lillis et al., 2012; Hudson and Grau, 2022). A substantial development of phenol-induced neurological symptoms and the concurrent recovery of ATPase activity after administering GABA_A_ergic drugs or phosphorylation blockers indicate that GABA_A_R/ATPase subtypes are the target of phenol action and can be used to investigate the effects of other phenols.

## 7 Conclusion

The first reports regarding the role of phenols in neuropsychiatric consequences and the establishment of the possible role of GABA_A_Rs in their manifestation have primarily focused on phenol derivatives. In this review, we examined the roles of phenols with different structures and neurobiological sequelae in GABA_A_R regulation. Due to the diversity and complexity of the structures of phenolic substances, they have different modes of action on GABA_A_Rs and elicit various diseases. We showed the similarities and differences between various phenol groups and how they can induce neurobiological changes ranging from negative action (e.g., SPs or BPs) to positive manifestations (e.g., PPs) in the CNS. The neuropsychiatric sequelae after exposure of GABA_A_Rs to phenols are confirmed by a number of studies, suggesting the dominant role of this type of pLGICs in the development of these diseases. In particular, simple phenols elevate GABA_A_R functional activity, whereas many natural polyphenols most often causes an inhibitory effect on receptor activity, albeit at varying efficiencies; however, mechanisms of action for most remain unclear. As shown above, the complex structures of natural chemicals imply the presence of diverse mechanisms regarding their interaction with GABA_A_R subunits, making it difficult to establish their molecular mechanisms of effect. However, although natural polyphenols are of particular interest, probably when establishing molecular causes in the development of neuropsychiatric disorders consideration primarily of simple phenol action on the GABA_A_Rs was preferable.

Indeed, the first step toward achieving effective GABA_A_R regulation using complex phenols (including natural phenolics) to treat the different neurobiological sequelae is to elucidate the specific sites that interact with simple phenols and the molecular events leading to positive conformational changes in their corresponding receptor subtypes. However, although our understanding of the interaction between phenol derivatives and GABA_A_Rs continues to increase, the molecular mechanisms underlying the interaction of the GABA_A_Rs to simple phenols (and primarily to phenol) remain largely elusive. In addition, crystal/cryo-EM and site-directed mutagenesis may not always identify the functional, critical, and complex phenols binding sites, as various compounds may interact with similar or distinct sites and elicit various functional consequences. Therefore, determining the mechanistic basis of phenol-GABA_A_R interactions at the molecular level is essential for elucidating the conformational rearrangements in the channel and could pave the way for the design of drugs with improved activity and target specificity.

One unique feature of GABA_A_Rs is their adoption of a phenol-dependent conformation. Recent data suggest that phenols mainly target the β subunit (primarily the β3 subunit) of GABA_A_Rs (Menzikov et al., 2021). Comparative analysis showed that phenols have different effects on the mediator-mediated responses by stabilizing or inhibiting different receptor conformations/states depending on the presence of bicarbonate. Although the exact molecular mechanism of phenol-receptor interactions remains unresolved, H-bond formation between the hydroxyl group of the phenol and the amino acid residues of the target subunits is likely; however, as shown above, other interactions are also possible.

The construction of phenol-specific structures (Leon et al., 2012) that target the GABA_A_R subunits is required to fully understand the molecular nature of the interaction of compounds with the GABA_A_ receptor. Using these model structures, along with a combination of functional (e.g., electrophysiology, fluorimetry) and structural (e.g., cryo-EM, MS, or NMR) studies, should eventually provide detailed insight into the mechanisms underlying phenol-GABA_A_R interactions. This will also allow us a better understanding of the pharmacological effects of other phenols that might modulate GABA_A_R conformation, which could result in a generate chemical templates for developing clinically important drugs. Finally, a mechanistic understanding of the interactions of phenol with the GABA_A_R-coupled ATPase during resensitization may have clinical significance for treating neurobiological sequelae. Indeed, this ATPase plays an essential role in GABA_A_R resensitization and the elimination of neuropsychiatric symptoms. However, discovering the mechanism whereby phenols modulate ATPase activity requires further detailed structural analyses the interactions of phenol with the GABA_A_Rs and specific enzyme inhibitors.
